# Sedative and Anticholinergic Burden as Predictors of Delirium in Hospitalized Older Adults

**DOI:** 10.1093/geroni/igaf122.2322

**Published:** 2025-12-31

**Authors:** Hadas Shachaf, Juliana Smichenko, Rami Klayn, Rabab Awad, Ron Oliven, Anna Zisberg

**Affiliations:** Bnai Zion Medical Center, Haifa, Hefa, Israel; University of Haifa, Haifa, HaZafon, Israel; Bnai Zion Medical Center, Haifa, Hefa, Israel; University of Haifa, Haifa, Hefa, Israel; Bnai Zion Hospital, HaifA, HaZafon, Israel; University of Haifa, Haifa, HaZafon, Israel

## Abstract

Delirium is a prevalent yet underdiagnosed acute cognitive disorder among hospitalized older adults, contributing to prolonged hospital stays, increased morbidity, and higher mortality rates. While medications with sedative and anticholinergic properties are known risk factors, their cumulative impact and potential as early indicators for delirium remain unclear. This study aims to assess whether sedative burden, anticholinergic burden, and polypharmacy can predict delirium in hospitalized older patients and serve as a trigger for early intervention. A retrospective cohort analysis was conducted on 260 patients (aged 65+) from three internal medicine wards. Patients were categorized based on delirium status (positive: n = 150, negative: n = 110) using RADAR and MOTYB screening tools. Sedative and anticholinergic medication burden was quantified using the Drug Burden Index (DBI), based on medication data from the first hospitalization day. Covariates included age, gender, illness severity (NEWS), comorbidities (Charlson Index), polypharmacy (≥5 medications), and physical status (Norton Scale). Logistic regression examined associations between medications burdens at admission and delirium risk. Results showed that patients screening positive for delirium had significantly higher sedative burden (OR = 2.93 CI [1.42-6.06], p=.004), while no significant differences were observed in anticholinergic burden. Physical status protected from (OR = 0.59 CI [0.51-0.69], p=.000) while polypharmacy (OR 3.63 CI [1.34-9.83], p = 0.011) and older age (OR = 1.12 CI [1.063-1.18], p=.000) significantly predicted delirium. These findings emphasize the need for systematic medication review upon hospital admission, with a focus on minimizing sedative load to mitigate delirium risk. Integrating delirium screening with pharmacological assessments may enhance early identification and intervention strategies, ultimately improving patient outcomes.

